# Physiological Effects of Mind and Body Practices

**DOI:** 10.1155/2015/983086

**Published:** 2015-06-10

**Authors:** Shirley Telles, Patricia Gerbarg, Elisa H. Kozasa

**Affiliations:** ^1^Patanjali Research Foundation, Haridwar, Uttarakhand 249405, India; ^2^New York Medical College, Valhalla, NY 10595, USA; ^3^Hospital Israelita Albert Einstein, 05601-901 São Paulo, SP, Brazil

Mind-body practices originated in ancient cultures to enhance physical, mental, and spiritual wellbeing. Interest in their use in treatment stems from increasing awareness of their therapeutic potential and from the need for approaches that are cost-effective and have lower risks for adverse effects compared to pharmacological and other conventional interventions [1–3]. Rigorous research is needed to identify effective mind-body techniques and their mechanisms of action, if they are to be integrated into mainstream medicine [4].

The quality of evidence for mind-body practices varies widely. Scales for rating the quality of research are based on pharmacological studies. Very often the research design applicable to conventional medicine does not apply to mind-body practices. The diversity and complexity of interventions further confound efforts at conventional meta-analysis. Current studies are developing better control interventions and implementing more rigorous methods [2]. The National Center for Complementary and Alternative Medicine (NCCAM) [5] states that “*Developing insight into biological, physiological effects and mechanisms of action of mind and body interventions is critically important in developing translational research tools to design and execute maximally informative clinical research.*” For evaluating mind-body research, modifications of the original CONSORT statement have been proposed and are being evaluated [6].

Studies of mind-body practices suggest numerous mechanisms of action at multiple levels, including gene expression at the cellular level; interactions among central brain regions with neuroplastic changes; and top-down and bottom-up feedback loops between the brain and the body, particularly via the nervous system, interoceptive communication, and circulating neurohormones [7–10]. Prominent among proposed mechanisms are the following: (a) repatterning of primary interoceptive and higher order homeostatic mechanisms; (b) improved central regulation of autonomic, psychologic, neurologic, immunologic, cardiorespiratory, and gastrointestinal functions; (c) reorganization within cortical and subcortical structures, interconnectivity adjustments among central regulatory networks, neurotransmitter changes, improved emotion regulation by higher centers, better interhemispheric balance, and enhanced cognitive function; and (d) modulation of epigenetic factors, such as growth factors or hormones, as well as extensive up- and downregulation of genes [11–13].

The manuscripts in this special issue include both research studies and reviews. For example, effects of mind and body practices on specific higher brain functions such as creativity are explored and with objective markers (pro-NGF levels). A study of 1297 adolescents documents the impact of mind and body practices on academic performance and cardiometabolic risk factors. In young musicians the effects of Qigong are reported to be an enhancement of proprioception and a reduction of anxiety-induced physiological changes. While these three studies focus on higher brain functions and proprioception, additional trials emphasize autonomic changes occurring with mind and body practices. For example, the effects of guided imagery on heart rate variability in students performing spaceflight emergency tasks are evaluated. In another study physiological feedback is matched with self-reported stress while participants perform short tasks. These studies involve healthy volunteers. A single clinical study examines the effect of mindfulness meditation on mood, quality of life and attention in adults with ADHD.

All four review articles are on meditation. An activation likelihood estimation (ALE) reports a co-ordinate based meta-analysis of neuro-imaging studies in meditations of different types. Another review examines the electrophysiological changes in different meditations based on evoked and event-related potentials. Two other reviews looked at the physiological and cognitive effects of meditation, considered as a ‘journey without a goal'; and the psychological and neural effects of mindfulness practice underlying its positive impact on health. An interesting comparative review explores the impact different meditation traditions have on the autonomic nervous system and on phasic or tonic changes in attention.

Identifying mind-body practices that show physiological or clear clinical benefits with appropriate biomarkers may lead to the refinement of certain techniques so that they become more efficient, less time-consuming, more effective, and better suited for the treatment of specific conditions. Also, mind-body practices can be used as noninvasive probes to explore fundamental neurophysiological processes and anatomic networks using brain imaging and other advanced technologies. Understanding the physiological changes, clinical benefits, adverse effects and contraindications associated with these practices will support the inclusion of mind-body treatments in mainstream medicine.

By highlighting studies with biological markers and physiological measures, this special issue is intended to further the understanding of mechanisms underlying the diverse effects of mind and body techniques.

*Shirley Telles* *Patricia Gerbarg* *Elisa H. Kozasa*
